# Performance evaluation of malaria microscopists working at rechecking laboratories in Ethiopia

**Published:** 2017-06-01

**Authors:** Abnet Abebe, Meseret Belayneh, Habtamu Asrat, Wondwossen Kassa, Andargachew Gashu, Adino Desale, Getnet Hailu, Tesfaye Mekonnen, Feven Girmachew, Achamyeleh Mulugeta, Ebise Abose, Dereje Yenealem, Abeba G Tsadik, Adisu Kebede, Gonfa Ayana, Kassu Desta

**Affiliations:** 1 Ethiopian Public Health Institute, Addis Ababa, Ethiopia; 2 Department of Medical Laboratory Sciences, College of Health Sciences, Addis Ababa University, Addis Ababa, Ethiopia

## Abstract

**Background:**

Microscopic diagnosis of Giemsa-stained thick and thin blood films has remained the standard laboratory method for diagnosing malaria. High quality performance of microscopists that examine blood slides in health facilities remains critically important.

**Materials and methods:**

A cross-sectional study was conducted to assess the performance of 107 malaria microscopists working at 23 malaria rechecking laboratories in Ethiopia. A set of 12 blood film slides was distributed to each microscopist. Data was collected and exported to SPSS version 20 for analysis. Chi-square, sensitivity, specificity, percent agreement, and kappa scores were calculated to assess performance in detecting and identification of *Plasmodium* species.

**Results:**

The mean age of the participants was 30 ± 5 yrs and most of them (54; 50.5%) were working at regional reference laboratories. Overall, the sensitivity of participants in detecting and identifying malaria parasite species was 96.8% and 56.7%, respectively. The overall agreement on detection and identification of malaria species was 96.8% (Kappa = 0.9) and 64.8% (Kappa = 0.33), respectively. The least accurately identified malaria parasite species was *P. malariae* (3/107; 2.8%) followed by *P. ovale* (35/107; 32.7%). Participants working at hospital laboratories had the highest percentage (72.3 %, Kappa=0.51) of accurate species identification. Study participants that had participated in malaria microscopy and quality assurance trainings were significantly better at quantifying parasite densities (P<0.001).

**Conclusion:**

The accuracy of parasite identification and quantification differed strongly between participants and expert microscopists. Therefore, regular competency assessment and training for malaria microscopists should be mandatory to assure proper diagnosis and management of malaria in Ethiopia.

## 1 Introduction

Malaria is a mosquito-borne infectious disease of humans and other animals caused by parasitic protozoans of the genus *Plasmodium* [1,2]. In humans, malaria is caused by *P. falciparum*, *P. malariae*, *P. ovale*, *P. vivax* or *P. knowlesi* [3]. Globally, an estimated 3.2 billion people in 97 countries and territories are at risk of being infected with malaria and developing disease, and 1.2 billion are at high risk. According to the latest estimates, 214 million cases of malaria occurred globally in 2015 and resulted in 438,000 deaths. Of these, 90% of all occurred in sub-Saharan Africa followed by the WHO Southeast Asia Region (7%) and the WHO Eastern Mediterranean Region (2%) [4]. Every minute a child on the African continent dies due to malaria [5]. In Ethiopia, malaria remains the leading cause of morbidity and mortality. Approximately 75% of the country is malarious, and 60% of the Ethiopian population (totalling 96.6 million in 2014) is at risk of malaria [6-8].

Correct diagnosis of malaria is vital to determine disease prevalence and incidence in order to evaluate the impact of malaria control interventions [9]. Training and competency assessment in malaria microscopy using already trained personnel and validated slides are important for evaluating, improving and maintaining high performance in malaria microscopy, and is also an opportunity to provide continuing education [10,11]. Many rechecking laboratories were established in different regions of Ethiopia to recheck the blood film slides examined at microscopic sites. Some of the rechecking laboratories are providing only referral services, so that they are not providing routine services on malaria microscopy. The competency status of the malaria microscopists that are working at rechecking laboratories is not known. Therefore this study aimed to assess the performance of malaria microscopists working at Malaria External Quality Assessment (EQA) Rechecking Laboratories in Ethiopia in terms of parasite detection, parasite species identification, stage identification and parasite quantification using light microscopy.

## 2 Materials and methods

### 2.1 Study setting and participants

**Figure 1:**
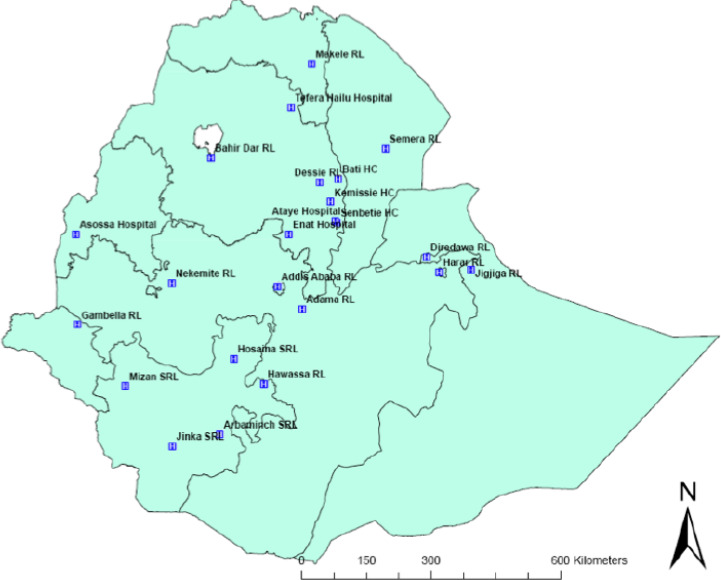
Malaria rechecking study sites in Ethiopia.

The study was conducted from February to May, 2015. Ethiopia is administratively structured with nine regional states and two city administration [12]. A total of 23 malaria rechecking laboratories were included in the study (Figure 1). All microscopists working at the malaria EQA rechecking laboratories were invited to participate in this study.

### 2.2 Slide preparation and validation

Whole blood (3-5 ml) was collected in EDTA tubes from volunteers above 18 years of age. A total of 200 blood film slides and Dried Blood Spot (DBS) were prepared from each donor. Six and two microliters were used to prepare a thick and thin blood film, respectively. Both thick and thin blood films were prepared on a single slide. The thin blood film was fixed with absolute methanol. After drying, both thin and thick films were stained for 30-45 minutes with 3% Giemsa working solution. Blood film for microscopic diagnosis of malaria was made semi-permanent by cover slip using Poly mount (toluene based, Cat. # 08381) and a cover glass. Six malaria microscopy experts (microscopists certified as level 1 by WHO after the examination of WHO AFRO external competency assessment) were involved in the validation process and each of them read 2 blood film slides from each donor. Discordant results among validators on species identification were verified by Polymerase Chain Reaction (PCR).

### 2.3 Administration of blood film slides

After validation, two sets of blood film (BF) slides with different values were given to study participants. The first set comprised a total of 10 BF slides; 2 negative slides, 8 positive slides (3 *P. falciparum* slides with different densities, 2 BF slides with *P. vivax*, 1 Mixed species slide of *P. falciparum* and *P. vivax*, 1 BF slide with *P. malariae,* and 1 BF slide with *P. ovale*). These BF slides were used for assessment of parasites detection, stage and species identification. The second set contained 2 positive slides; 1 *P. falciparum* BF slide with a parasite density of 1891 parasites/μL (with a range of 1419-2363 parasites/μL) and 1 *P. falciparum* BF slide with a parasite density of 50,659 parasites/μL (with a range of 37990-63323 parasites/μL) were used for the assessment of quantification performance. Based on WHO recommendation, quantification results that deviated ±25% from the mean calculated by expert readers was considered as correct quantification result. A total of 120 minutes (10 minutes per BF slide) were allocated for the exercise [13].

### 2.4 Questionnaire

A structured and standardised questionnaire, which was used to obtain information about participating facilities and microscopists, was distributed amongst the participants. The questionnaire addressed components like age, sex, educational background, type of health facility, in service training, work experience, routine practice of the professional, and number of slides rechecked per person. The data was collected by those that had good experience in malaria microscopy at Ethiopian public health institute laboratories.

### 2.5 Data management and quality assurance

The quality of the blood film slides was checked by reference readers against WHO standards before distribution to malaria microscopists, and the results were critically assessed for completeness. Pre-testing of each questionnaire type was done with 5% of the study population. Intensive supervision was done during data collection.

### 2.6 Statistical analysis

Data was collected and entered into Microsoft Excel sheets and exported to software SPSS version 20 for analysis. Chi -square (for categorical data), sensitivity, specificity, percent agreement, and kappa scores were calculated to assess laboratory professionals’ performance in detecting and identification *Plasmodium* species. Based on WHO classification, microscopists were considered as: ‘In training’ - when the agreement with the expert reader in detection with species identification of malaria parasite was less than 70%; ‘Advanced’ - when the agreement was greater than or equal to 70% but less than 80%; ‘Reference’ - when the agreement was greater than or equal to 80% but less 90%; and ‘Expert’ - when the agreement was greater than or equal to 90% [13]. Kappa values were calculated to determine the strength of an agreement. Based on the calculation the strength was classified as: < 0.20 slight agreement, 0.21–0.40 fair agreement, 0.41–0.60 moderate agreement, 0.61–0.80 substantial agreement, or 0.81–0.99 almost perfect agreement [14]. A percent agreement score >80% is a passing point for external quality assessment based on WHO and national guidelines [13,15].

### 2.7 Ethical clearance

This study was done after obtaining approval from the ethical clearance committee of Addis Ababa University College of Health Sciences, Department of Medical Laboratory Sciences, departmental research and ethics review committee. Information about the study was provided to all malaria microscopists involved in the study and to all concerned bodies at the study sites. All participants were assured about the confidentiality, protection and anonymity of the data. Written informed consent was obtained from voluntary study participants before conducting the study.

## 3 Results

From a total of 129 malaria microscopists that were engaged in rechecking blood film slides, 107 (83%) participated in the study. The mean age (± SD) of the participants was 30 ± 5 yrs and 90 (84.1%) were men (Table 1).

**Table 1:** Characteristic of malaria microscopists working on malaria microscopy at malaria EQA rechecking laboratories in Ethiopia (n=107).

Characteristic	n	%
Age (years)		
20-30	68	63.6
31-40	37	34.6
>41	2	1.9
Sex		
Male	90	84.1
Female	17	15.9
Educational status		
Diploma	25	23.4
Degree	63	58.9
MSc and above	19	17.8
Experience with malaria microscopy		
< 2 Years	4	3.7
2-5 Years	43	40.2
> 5 Years	60	56.1
Place of work		
Health centre laboratory	16	15.0
Hospital laboratory	22	20.5
Sub-regional laboratory	15	14.0
Regional reference laboratory	54	50.5
Previous participation in malaria microscopy and QA training		
Yes	83	77.6
No	24	22.4
Number of BF slides rechecked per year		
≤150	56	52.3
151-300	39	36.4
301-450	6	5.6
≥451	6	5.6

Based on WHO classification, 78 (72.9%) of the participants were ‘in training’ with respect to species identification. Only 11(10.3%) participants quantified both BF slides correctly while 34 (31.8%) used a non-recommended quantification system (Table 2). From a total of 1070 blood film slides that were distributed for detection, 1036 (96.8%) were correctly reported for the presence or absence of malaria parasites and from a total of 856 positive blood film slides only 486 (56.8%) were reported correctly in terms of species identification. Of 214 BF slides that were distributed for quantification, only 63(29.4%) were quantified correctly.

**Table 2:** Overall performance in terms of parasite species identification, stage identification and parasite quantification according to WHO classification (n=107).

Performance classification	Frequency	%
% Agreement on species identification		
≥ 90 (expert)	0	0
≥ 80 - 90 (reference)	10	9.3
≥ 70 - 80 (advanced)	19	17.8
< 70 (in training)	78	72.9
% Agreement on stage identification		
≥ 90	33	30.8
≥ 80 - 90	35	32.7
≥ 70 - 80	27	25.2
< 70	12	11.2
Performance on parasite quantification		
Quantified both BF slides correctly	11	10.3
Quantified one BF slide correctly	45	42.0
Missed both	17	15.9
Used non-recommended quantification system	34	31.8

The overall agreement on detection of malaria parasites was 96.8% (Kappa = 0.9), which is ‘perfect agreement’ while on identification of malaria species it was 64.8% (Kappa = 0.33) which is a ‘fair agreement’ (Table 3). False positive rate (negative BF slides reported as positive) and false negative rate (positive slides reported as negative) were 0.84% (7/836) and 11.5% (27/234), respectively.

**Table 3:** Overall sensitivity, specificity and agreement between participants and expert accuracy in detecting and identifying parasite species.

Participant reader		Expert reader			Sensitivity	Specificity	Agreement	Kappa
		Positive	Negative	Total				
Parasite detection	Positive	829	7	836	96.8%	96.7%	96.8%	0.9
	Negative	27	207	234				
	Total	856	214	1070				
Species identification	Positive	486	7	493	56.7%	96.7%	64.8%	0.33
	Negative	370	207	577				
	Total	856	214	1070				

**Figure 2:**
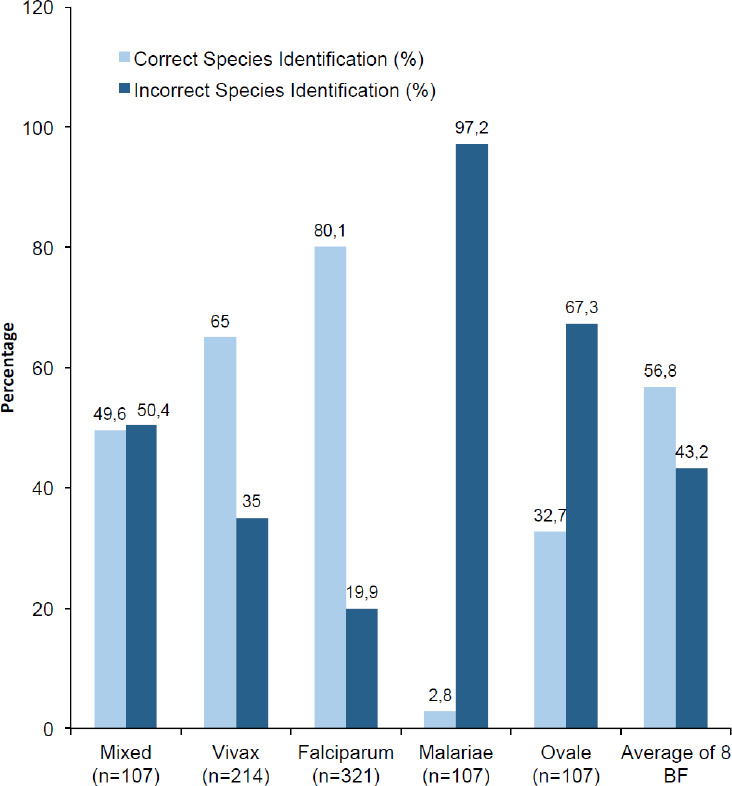
Accuracy of malaria microscopists in determining malaria parasite species (8 different known positive BF slides).

The performance of participants in species identification showed that 80.1% of BF slides with *P. falciparum* but only 2.8% of BF slides with *P. malariae* were identified correctly (Figure 2). A total of 64 (61.5%) BF slides with *P malariae* were identified wrongly as *P. falciparum*, 42 (58.3%) BF slides with *P. ovale* were identified wrongly as *P. vivax*, and 37 (34.6%) of cases with mixed (*P. falciparum* and *P. vivax*) BF slides were reported only partially correctly as *P. falciparum*.

Out of 107 participants, 97 (90.7%) scored <80% overall, which indicates failure based on WHO and national guidelines [13,15]. The number of participants that scored a pass mark (≥80%) was higher among participants with MSc level education (6; 31.6%), or participants with more than 5 years work experience (9; 15%) followed by 2-5 years experience (1 participant; 2.3%), respectively (P=0.75). The number of participants that scored ≥80% was higher among participants that had received malaria microscopy and QA training 10 (12.1%) while all participants that had not received training on malaria microscopy and QA scored below 80% (P=0.074).

Participants working at hospital laboratories had the highest percentage agreement with expert readings (72.3%, Kappa=0.51) while regional reference laboratories had the lowest (61.7%, Kappa=0.46; Table 4).

**Table 4:** Agreement between participants (n=107) and expert reader in identification of malaria species, grouped by type of health facility.

Variable		Participant reader	Expert reader			Sensitivity	Specificity	Agreement	Kappa
			Positive	Negative	Total				
Type of health facility	Regional reference laboratory	Positive	230	5	235	53.2%	95.4%	61.7%	0.46
		Negative	202	103	305				
		Total	432	108	540				
	Sub regional laboratory	Positive	66	0	66	55.0%	100%	64%	0.47
		Negative	54	30	84				
		Total	120	30	150				
	Hospital laboratory	Positive	115	0	115	65.3%	100%	72.3%	0.51
		Negative	61	44	105				
		Total	176	44	220				
	Health centre laboratory	Positive	75	2	77	58.6%	93.8%	65.6%	0.49
		Negative	53	30	83				
		Total	128	32	160				

Quantification performance of malaria microscopists working at sub-regional laboratories was 73.3% which was higher than malaria microscopists performance from other malaria rechecking laboratories (P=0.003). Study participants that participated in malaria microscopy and QA training had a better performance on quantification than those who had not received training (P<0.001) (Table 5).

**Table 5:** Relationship between quantification performance of malaria microscopists (n=107) and work experience, educational status, types of health facility and training.

Variables	Quantified at least one BF slide (%)	Missed both BF slides (%)	Chi-Square	df	p-value
Educational Status					
Diploma	11 (10.3)	14 (13.1)	1.0	2	0.607
Degree	34 (31.8)	29 (27.0)			
MSc or higher	11 (10.3)	8 (7.5)			
Work Experience (years)					
< 2	2 (1.9)	2 (1.9)	0.4	2	0.823
2-5	21 (19.6)	22 (20.6)			
> 5	33 (30.8)	27 (25.2)			
Place of Work					
Health centre laboratory	6 (5.6)	10 (9.3)	14.2	3	0.003
Hospital laboratory	5 (4.7)	17 (15.9)			
Sub regional laboratory	11 (10.3)	4 (3.7)			
Regional reference laboratory	34 (31.8)	20 (18.7)			
Participation in malaria microscopy and QA training					
Yes	54 (50.5)	29 (27.1)	24.0	1	<0.001
No	2 (1.9)	22 (20.5)			

## 4 Discussion

Malaria remains a leading cause of morbidity in Ethiopia [7]. Microscopy of Giemsa-stained thick and thin blood films is the gold standard method for diagnosis of malaria. Competency assessment is one of the methods to verify microscopists’ competency to perform laboratory tests and produce accurate, reliable and timely results. In the current study an agreement between participants and malaria microscopy expert readers in the detection of malaria parasites was 96.8% (Kappa=0.9) which was relatively higher when compared with the study conducted in Hawassa town, Ethiopia which was 88% (Kappa=0.67) [16]. Based on WHO recommendation 78 (72.9%) participants were ‘in training’ in the current study while only 17 (23.6%) were rated as ‘in training” in the study conducted in Hawassa town [16]. The reason for this may be lack of providing routine service; which is used to develop diagnostic skills for malaria parasite detection and identification, and/or lack of regular training on malaria microscopy.

Both the sensitivity (96.8%) and specificity (96.7%) of microscopists in this study were higher than in the study conducted in Hawassa town which was 82% and 96.5% [16], in USA it was 92% and 90% [17], and in a study from Zambia it was 88% and 91% [18], respectively. The higher sensitivity in the current study regarding the detection of parasites indicates that there was a low rate of false negative results, which means that misdiagnoses of true infections was low. This can help an individual to take appropriate treatment in line with malaria.

Overall, strength of agreement in identification of different species of malaria in the current study (Kappa = 0.33) was lower than the study conducted in North Gondar (Kappa = 0.47) [19] and Hawassa town (Kappa=0.63) [16]. The number of participants that failed to correctly speciate *P. falciparum* (19.9%) in the current finding was slightly higher than a similar study conducted in Hawassa, where this was about 18% [20] but lower than a survey conducted in Canada 27% [21]. Blood film slides with *P.ovale* which were identified correctly (32.7%) was higher than the study conducted in North Gondar, northwest Ethiopia in which no microscopist correctly identified *P. ovale* [22]. Low performance on species identification may be due to the fact that some participants did not spend sufficient time to examine the slides, or possibly due to lack of regular training. Lack of correct species identification may lead to incorrect administration of first line treatment. For example the recommended first-line treatment of all clinically and parasitologically-diagnosed uncomplicated *P. falciparum* malaria in Ethiopia is an artemisinin-based combination therapy (ACT) called artemether-lumefantrine (AL) while the first line treatment for *P. vivax* is oral chloroquine [6]. Correct species identification can be used to treat an individual with an appropriate first line drug and used to prevent drug resistance.

In our study, the false positivity rate (negative BF slides reported as positive) was 0.8% which was lower than the study conducted in Canada (2.0%) [21], 6.9% reported in Hawassa town [16], 7% reported in USA [17], 12% reported in Hawassa, Ethiopia [20], and 19% reported in Democratic Republic of Congo [23]. These false positive results could lead to unnecessary treatment with antimalarial drug or a delayed diagnosis of the true cause of illness and misleading the clinician from considering other causes of fever and disease. The false negativity rate (positive slides reported as negative) in our study was 11.5% which was higher than 10.1% reported in Democratic Republic of Congo [23] and 3% reported in USA [17]. False negativity can lead to delayed treatment, development of serious complications and possibly death or exposure to unnecessary treatment with other (non anti-malaria) drugs.

Quantification performance of malaria microscopists working at sub-regional laboratories was 73.3%, which was higher than malaria microscopist performance from other malaria rechecking laboratories (P=0.003).This might be because most participants that were working at sub-regional laboratories were trained on malaria microscopy and QA training. Study participants that were trained on malaria microscopy and QA performed significantly better on quantification (P<0.001). Correctly-quantified blood film slides in our study were 31.3%, which was lower than 81% of correct quantification reported from the USA [17]. Participants that used a non-recommended quantification (+) system in this study was 31.8%, which was lower than the study conducted in Democratic Republic of Congo in which 68.6% of the participants used this quantification system [23]. Parasite quantification using non-recommended system may be due to lack of updated information (lack of training), or lack of awareness of the advantage of the quantitative system over a semi quantitative (+) system, or it might be due to lack of commitment to adhere to specified time for counting parasite with the recommended quantification system. This is important because measuring parasite density can be used to monitor patient response to treatment and to study drug efficacy.

## 5 Conclusions

Even though the performance of participants was good in detection of malaria using microscopy their agreement with expert microscopists identification of different malaria species and the quantification of parasite densities were very low. Most participants did not identify *P. malariae* and *P.ovale* correctly. The ability to identify true positives was lower amongst participants that worked at regional reference laboratories. Participants that were not trained on malaria microscopy displayed very poor performance in parasite quantification. Quantification performance of microscopists working at health centre or hospital laboratories was very low. Therefore, to fill the identified gaps, all stakeholders at all levels have to work on the implementation of regular competency assessments and implement training policy. Demonstration BF slides used for malaria microscopy training have to comprise *P. malariae* and *P.ovale*. Regions which are using or which are going to use health centre and hospital laboratories as rechecking sites have to evaluate the performance of laboratories using all external quality assessment methods and have to conduct competency assessment for malaria microscopists that are working at those rechecking sites.
